# Research on the Method of Detecting TPN-Labeled Tumor Cells in Pleural Effusion Based on the Microfluidic Chip

**DOI:** 10.3390/mi15080981

**Published:** 2024-07-30

**Authors:** Xiaoyi Xun, Shuang Song, Yiran Luan, Xiaoyue Long, Peilan Zhang, Yuqun Zheng, Xuguo Sun

**Affiliations:** 1 School of Medical Laboratory, Tianjin Medical University, Tianjin 300203, China; xunxiaoyi2000@163.com (X.X.); luanyiran1999@163.com (Y.L.); zhengyuquntmu@163.com (Y.Z.); 2 National Clinical Research Center for Cancer, Beijing 101399, China; 17633067069@163.com; 3 Department of Laboratory, Tianjin Huanhu Hospital, Tianjin 300350, China; 18632332447@163.com (X.L.); peilanzhng@sina.com (P.Z.)

**Keywords:** MPE, tumor cells, microfluidic chip, TPN

## Abstract

The clinical diagnosis of a malignant pleural effusion (MPE) is still based on the detection of tumor cells in the pleural effusion. The question of how to improve the efficiency and accuracy of detecting an MPE still remains. This study explores the use of microfluidic technology to concentrate cells in an MPE and achieved the detection of the cell marker TPN in the microarray capture area. TPN is a mitochondria-specific bio-probe that can identify tumor cells on the basis of differences in the mitochondrial potential. First, we designed a microfluidic chip to analyze its performance. The results show that when the total flow rate of the injected chip was 12 mL/h and the volume ratio of cell separation liquid to cell suspension was 1:1, the target cells (A549, MCF-7, and Hela) were enriched and the purity was improved to 98.7–99.3%. Finally, an MPE from cancer patients was used to detect the chip’s ability to isolate and enrich tumor cells. Furthermore, the fluorescent identification of the TPN within the tumor cells was simultaneously achieved on the microfluidic chip. In conclusion, the potential to improve the efficiency of the clinical diagnosis of MPEs is provided by the chip structure and analysis conditions explored in this study.

## 1. Introduction

A malignant pleural effusion (MPE) is a pathology in which a malignancy causes an abnormal increase in the fluid in the pleural cavity. Primary tumors and the secondary spread of diseases such as malignant mesothelioma of the pleura [1], lung [2], and breast [3] and lymphoma [4] and gastrointestinal tumors can complicate malignant pleural effusions. There are numerous types of background cells in the MPE [5,6], including erythrocytes, leukocytes, and mesothelial cells, which interfere with the detection of tumor cells. At present, the sensitivity of the clinical cytology examination of MPEs can reach about 60% [7]. Therefore, research on detection modalities to improve the isolation, concentration, and identification of tumor cells in pleural effusions will help improve the diagnostic efficiency of MPEs.

Microfluidic technology has been shown to be effective in concentrating and isolating cells in suspension [8,9,10]. Microfluidic technology has become increasingly popular in recent years for detecting malignant plasmapheresis [11,12,13]. Initially, researchers focused on the structure of microfluidic chips to passively separate and purify tumor cells from body fluids based on hydrodynamics. The purity of tumor cells from MPEs has been reported to increase 6–65-fold [14,15]. However, due to the complexity of cell types in MPEs as well as the scarcity of tumor cells compared to other non-tumor cells, how to concentrate tumor cells in MPEs has become a need. In a recent study, Ni et al. [16] reported an inertial microfluidic concentrator-based chip for concentrating the cells in an MPE, which increased the density of tumor cells at the exit of the chip. In addition, a study by Zhao et al. [17] used an integrated microfluidic device as a whole to analyze single cells. In their study, the separated cells were removed from the first chip of the device and then injected into the second stage of the single-cell analysis chip, and then the tumor cells on the chip were identified by immunohistochemistry. Finally, there is no method for MPEs that achieves the integration of the isolation and concentration of pleural effusion cells while detecting tumor cell-specific biomarkers.

With the development of biomedicine, there are specific biomarkers [18] found in tumor cells, including cell-surface biomolecules [19], intracellular biomolecules, metabolic enzymes [20], and aptamers [21]. Detecting tumor-specific markers on the cytomorphology has been reported to increase the diagnostic accuracy and sensitivity of cancer [17,22]. This makes it challenging to develop an assay that comprehensively screens tumor cells and detects tumor markers.

Recently, 1-(4-azidopropyl)-4-methylpyridinium hexafluorophosphate (TPN) was reported as a mitochondria-specific bio-probe [23] that contributes to the accuracy of tumor-cell identification. It has been proposed that the aggregation-caused quenching (ACQ) phenomenon [24] occurs when multiple organic fluorescent molecules are aggregated. TPN, as a luminescent source with aggregated-induced emission (AIE) activity [25], can penetrate into cells without killing them. Additionally, it can aggregate on the mitochondrial membrane of the cell, inducing the occurrence of yellow fluorescence, which is the opposite of the phenomenon of ACQ. An increased metabolic activity of tumor cells leads to a rise in the mitochondrial membrane potential [26,27]. A study comparing the difference in fluorescence intensity between TPN-labeled tumor cells and non-tumor cells suggests that TPN can achieve the function of identifying tumor cells [23]. This probe has been shown to discriminate tumor cells in MPEs.

In this study, we propose a microfluidic chip for the isolation and concentration of tumor cells in MPEs. Hydrodynamics is used to differentiate between nucleated cells and erythrocytes in pleural effusions. The different cells were each driven into different microfluidic channels on the chip, and the tumor cells were captured in specific areas. Fluorescence was used to simultaneously visualize the TPN in tumor cells. Therefore, a method for the specific identification of tumor cells on microfluidic chips was established, with the aim of providing a new diagnostic method for clinical medicine.

## 2. Materials and Methods

### 2.1. Experimental Materials

#### 2.1.1. Cultured Cell Lines

Lung, breast, and ovarian cancers are some of the most common cancers that develop thoracic metastases leading to malignant pleural effusions. Therefore, human lung cancer cells, A549, breast cancer cells, MCF-7, and ovarian cancer cells, Hela, were selected for the preparation of cell suspension. Additionally, the human mesothelial cell MET-5A and human WBCs were used as non-cancer cell lines for this study. For cell suspension preparation, A549, MCF-7, Hela, and Met-5A (Cell Resource Center, Shanghai Institutes for Biological Sciences, Chinese Academy of Sciences) were cultured as tumor cells and normal cells in pleural effusions, respectively. The tumor cells and Met-5A were cultured in a DMEM medium and an RPMI-1640 medium (Thermo Fisher Scientific Inc., Waltham, MA, USA) containing a 10% fetal bovine serum and 1% penicillin/streptomycin (Thermo Fisher Scientific Inc., Waltham, MA, USA). The cells were cultured at 37 °C in an atmosphere with 5% CO_2_. The WBCs were isolated from clinically healthy blood samples.

#### 2.1.2. Clinical Pleural Effusion and Different Types of Pleural Effusion Cells

Pleural effusion samples were collected from patients diagnosed with lung cancer, according to the guidelines for the clinical diagnosis and treatment of lung cancer. A total of 15 cases of MPE were collected (Table S1). Before the experiment, the pleural effusion cell count was adjusted to 2 × 10^6^ cells/mL using a Phosphate Buffer Solution (PBS) (Golden Clone (Beijing) Biotechnology Co., Ltd., Beijing, China).

#### 2.1.3. Microfluid Chip

The microchannel structure of the chip was designed and drawn using the AutoCAD software 2020. A PDMS polymer was used as the material for microfluidic chip fabrication. The chip fabrication process involves photolithography, which includes pre-treatment, leveling, pre-baking, exposure, post-baking, and development. The mold is created at this point. Then, a liquid polymer, PDMS, was poured on the mold, and the cured PDMS was peeled off the mold and sealed with a slide to obtain the microfluidic chip used in this study.

The chip consists of a helical channel measuring 310 μm in width and 63 mm in length, an expansion channel measuring 2250 μm in width and 2800 μm in length, and two capture areas. There were 2310 trapping sites in the larger area (Capture area 1) and 273 trapping sites in the other (Capture area 2). The horizontal distance between trapping sites is 40 μm, while the vertical distance is 95 μm. Each trapping site has a top diameter of 14 μm and a bottom diameter of 5 μm (Figure S1).

### 2.2. Methods

#### 2.2.1. Cell Labeling with the TPN Probe

The TPN probe was incubated with the cell line to be analyzed for 5 min in the absence of light and centrifuged at 300× *g* for 5 min to discard the supernatant. The suspension of cells was obtained after resuspension in the PBS. After the concentration of the pleural fluid cells was adjusted, the pleural fluid cells were labeled with TPN under the same conditions described above.

#### 2.2.2. Cell Staining with DAPI

Cultured tumor cells were fixed with 4% paraformaldehyde for 10 min; after that, they were centrifuged at 300× *g* for 5 min, and then the supernatant was discarded. The PBS was resuspended and a 30% DAPI (1 mg/mL) staining solution was added, and the samples were incubated for 15 min, centrifuged at 300× *g* for 5 min, and finally resuspended in the PBS to be used for the experimental study.

#### 2.2.3. HE (Hematoxylin–Eosin) Staining of Cells

After the pleural effusion cells were coated on the slides, they were fixed with 95% ethanol, then fixed with a hematoxylin-staining solution for 10 min, and finally re-stained with an eosin-staining solution for 1–2 min and sealed with Neutral Gum.

#### 2.2.4. Preparation of Cell Suspension

Tumor cells were treated with a DAPI solution. Cell suspensions were prepared by mixing diluted healthy blood samples with cultured tumor cell lines.

The total cell concentration was adjusted to 2^10^6^/mL, with tumor cells accounting for 30% of the total number of cells. After adjusting the concentration of the blood cells to 2.8^10^6^/mL and tumor cells to 1.2^10^6^/mL using a cell counter, the resulting suspension was checked by mixing the diluted blood sample with the tumor cell suspension in equal proportions and then passing the resulting suspension through a cell counter.

#### 2.2.5. Analysis of Fluorescence Intensity

Fluorescence microscopes (ECLIPSE Ni-U and ECLIPSE Ti-U, Nikon, Tokyo, Japan) were used to observe the TPN-labeled cells. For the TPN-labeled cells before and after chip processing, an analysis of fluorescence results using a fluorescence spectrophotometer (F-380, Gangdong, Tianjin, China) and the Image J 2 software was conducted.

#### 2.2.6. Statistical Analysis

A statistical analysis was performed using the Graphpad Prism 10.1.2 software. One-way ANOVA and two-way ANOVA were used to analyze the differences in the expression of TPN in the cells. One-way ANOVA was used to compare the TPN fluorescence intensity differences between cell lines. Two-way ANOVA was used to compare the effects of on-chip and off-chip environments and cell species on the TPN fluorescence intensity. Statistical insignificance was defined as ns. Differences were considered significant only if *p* < 0.05 (* indicates *p* < 0.05, ** indicates *p* < 0.01, and *** indicates *p* < 0.001).

## 3. Results and Discussion

### 3.1. The Structure and Principle of the Microfluidic Chip

**Figure 1:**
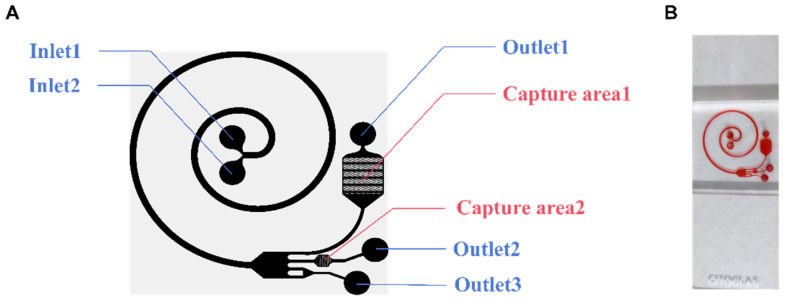
The structure of the microfluidic chip. (**A**) Schematic diagram of the microfluidic chip. (**B**) Photograph of a single chip filled with dye.

In this research, a microfluidic chip (Figure 1B) was designed to analyze the applicability of this composite structure for detecting tumor cells in pleural effusions. The microfluidic chip has two parts: the cell separation area and the capture area. The chip has two inlets (Inlet 1 and Inlet 2), two capture areas (Capture area 1 and Capture area 2), and three outlets (Outlet 1, Outlet 2, and Outlet 3) (Figure 1A). As the cell suspension enters the cell-separation area, the cells are subjected to a shear-induced lift (F_SL_), which moves them away from the center of the fluid toward the wall, while the cells near the wall are subjected to a wall-induced lift (F_WL_), which moves them away from the wall. And the F_WL_ and F_SL_ are collectively referred to as the net inertial lift (F_L_) [28]. Cells in a spiral channel will flow through the center of the channel faster than the outside of the channel. Cells will tend to move toward the outside of the track due to a centrifugal force. Due to the conservation of mass, there are two reverse vortices at the top and bottom of the channel, called Dean flows [29]. Cells will then be subjected to another Dean force (F_D_). When the F_L_ equilibrates with the F_D_, the cells in the track reach their respective equilibrium positions. For the larger projects, the F_L_ dominates to keep the cells close to the inside of the channel. The smaller ones move to the outside of the channel under the pull of the F_D_. The cells are thus initially separated. Dilated channels work by increasing the distance between cells of different volumes. From the narrow spiral channel to the wide expansion channel, cells of different volumes are further separated.

Ideally, when injecting cell suspension from Inlet 2 and cell separation from Inlet 1, red blood cells (RBCs) and nucleated cells are first separated, and then, the RBCs are removed from the chip along the outside of the channel. The remaining cells then follow the channel into another part of the chip, known as the capture area. There are two capture areas where solids are trapped as the fluid passes through. Without any additional requirements, the chip completely relies on continuous inertial microfluidics to achieve a high concentration and purification of tumor cells.

### 3.2. Exploration of Microarray Operating Parameters

**Figure 2:**
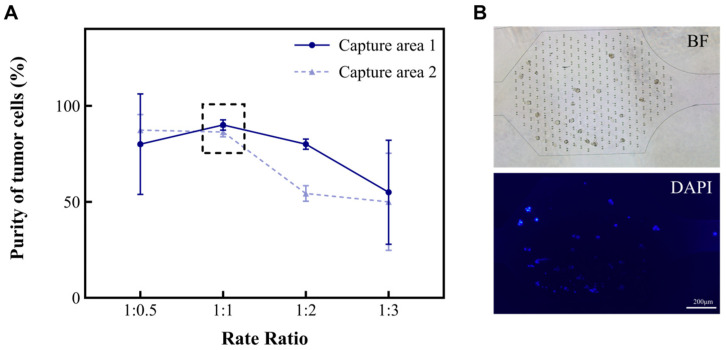
The relationship between the volume ratio of cell suspension to separation solution and the purity of A549 in the capture areas. (**A**) The purities (%) of A549 in the two capture areas of the microchip when the ratios between A and B are 1:0.5, 1:1, 1:2, and 1:3, respectively. (**B**) Cancer cells in the capture areas. Microscopic images including the bright field (BF) and fluorescence field (DAPI).

The different types and amounts of cells (such as red blood cells, white blood cells, and tumor cells) found in MPEs are related to the diseases that patients suffer from. In this study, we developed a microfluidic chip for analyzing MPEs. To investigate the characteristics of the device for the separation and concentration of pleural effusion cells, an A549 suspension and a separation solution were injected into the microfluidic chip at the flow rate ratios of 1:0.5, 1:1, 1:2, and 1:3, respectively. The volume of the cell suspension was kept at 2 mL and the total flow rate at 6 mL/h. The purities of A549 in the capture areas of the chip were then calculated separately (Figure 2A). Purity is defined as the ratio of tumor cells to the total number of cells in the capture area (Formula (1)). As shown in the results, the purity of A549 in both Capture area 1 and 2 tended to be 95% when the ratios of cell suspension to separation solution were 1:0.5 and 1:1. However, repeated experiments showed that it was more stable at 1:1. Hence, the ratio of 1:1 was chosen for subsequent studies.

**Figure 3:**
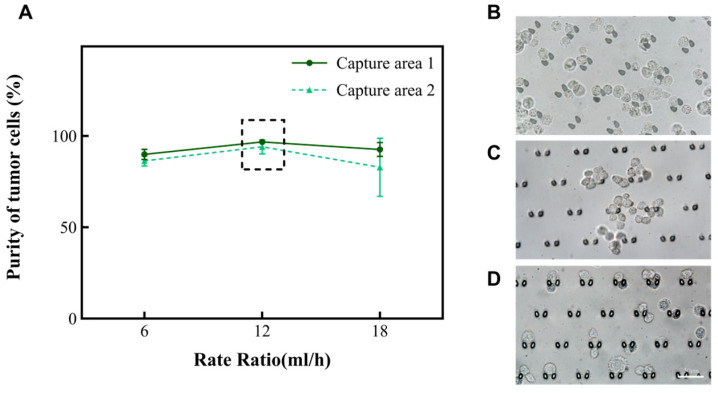
The relationship between the total rate and the purity of A549 in the capture area with the volume ratio of cell suspension to separation solution of 1:1. (**A**) The purity of A549 in the two capture areas when the total rate is 6 mL/h, 12 mL/h, and 18 mL/h, respectively. (**B–D**) Images of A549, Hela, and MCF-7 in the capture area when the total rate is 12 mL/h, and the ratio of A and B volumes is 1:1.

According to the principle of hydromechanics, the structure of the microfluidic chip helps the fluid to drive cells in the suspension in different directions. Smaller cells (like red blood cells and small white blood cells) will move along the outer wall of the channel, while larger cells (like large white blood cells, mesothelial cell and tumor cells) will move along the inner wall of the channel toward the capture areas. Subsequently, we altered the total rate at which the chip operated to 6 mL/h, 12 mL/h, and 18 mL/h to explore the purity of A549. The results are shown in Figure 3A. Nevertheless, when the rate of the injected chip reaches 18 mL/h, there is the phenomenon of a deformation of the cells due to the effect of an increased pressure. Fewer cells enter the capture areas at a rate of 6 mL/h (Figure 2B). In summary, the microfluidic chip performed effectively when the total rate of the injection device was 12 mL/h and the ratio of the rate of suspension to separation solution was 1:1. At this time, the purity of A549 was enhanced from 30% to 94.1~96.8% in the captured areas (Table 1). Subsequently, this research also analyzed the performance of this chip on two other types of tumor cells (MCF-7 and Hela). It was found that the purity of MCF-7 could be enhanced to 98.5~99.3%, and the purity of Hela cells could be enhanced to 98.1~98.7%. Moreover, the cell clusters were also able to be intercepted in the capture area of the microfluidic chip (Figure 3B–D). To ensure the accuracy of the results, the tumor cells in the cell clusters were also counted individually under a microscope.

Initially, tumor cells (A549) accounted for 30% of the inlet (Figure S2A). However, they did not reach 100% in either Capture area 1 or 2. Most of these RBCs just pass by due to their small size and leave through Outlet 1 and 2 (Figure S2B). Only a few will be left together in the capture areas due to an adhesion to the tumor mass. In this way, our device allows for the efficient separation of tumor cells.

The volume of each sample detected by the clinical pathologic cytology of pleural fluid is approximately 10–20 μL. In addition, because of the small number of tumors, locating tumor cells under a microscope and identifying and characterizing their morphology is an enormous amount of work. Not only is a great deal of clinicopathologic experience required of the microscopist, but the task is also very subjective. The microchip mentioned in this study is capable of concentrating tumor cells. The microfluidic chip increased the purity of the tumor cells by 3.15 to 3.31 times (Formula (2)), which will contribute to improved diagnostic sensitivity while diminishing the rate of missed diagnoses.

The formulas are as follows [14,30]:

(1) $$Purity\left(\%\right)=Tumor\;cells/All\;cells$$

(2) X=Purity(%)/Original purity(30%)

### 3.3. Observation of the Performance of Mitochondrial Labeling of Specific Tumor Marker TPN

As a synthetic precursor of a fluorescent probe for the in vivo imaging of apoptosis, the structure of TPN first came into view [31]. This compound was then used for the real-time imaging of cell behaviors in living organisms, as observed in cultured cells and in live zebrafish. It was found to selectively bind to mitochondria and emit fluorescence [32]. Researchers later labeled several tumor cell lines and leukocytes with TPN to measure their fluorescence expression by flow cytometry. Significant differences in fluorescence were found between the cancerous and non-cancerous cell lines. And using this property, TPN-labeled tumor cells were recovered from blood and pleural effusions, demonstrating the feasibility of TPN for tumor-cell identification [23]. As a cellular metabolic marker, TPN detects a wide range of tumor cell types. Because it mainly relies on the mitochondrial potential difference to label the cells, it avoids the leakage of the immunolabeling detection of cells when the cell-surface markers are under-expressed or non-expressed. Basic research on the TPN labeling of tumor cells has shown that it is effective in vitro, but no systematic study of how it works in tumor cells on microchips has been reported.

**Figure 4:**
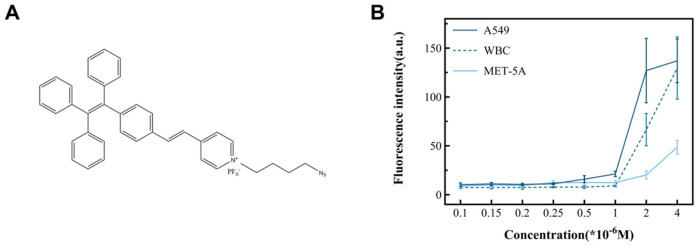
The structure of TPN and its ability to identify tumor cells. (**A**) The chemical structure of TPN. (**B**) Fluorescence intensity changes in cell lines incubated with different TPN concentrations, including cancer (A549) and non-cancer cell (WBC and MET-5A) lines.

**Figure 5:**
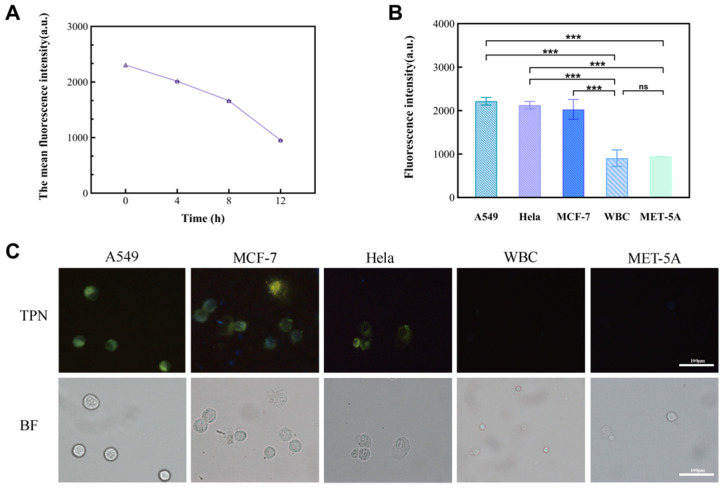
The significance of the fluorescence intensity changes observed in different cell lines. (**A**) Fluorescence intensity of TPN-labeled A549 cells over time (0–12 h). (**B**) Comparison of fluorescence intensity between tumor cell lines (A549, MCF-7, and Hela) and non-tumor cell lines (MET-5A and WBC) (*n* = 9, *** *p* < 0.001, Statistical insignificance was defined as ns). (**C**) Images of five TPN-labeled cell types (BF, bright field; TPN, fluorescence field).

In this study, cancer cell lines (A549, MCF-7, and Hela) and non-cancer cells (MET-5A and WBCs) were used as subjects to observe the performance of TPN labeling on different cells (Figure 4B). The results show that there was a significant difference in the fluorescence intensity between the cancer and non-cancer cell lines at a TPN concentration of 1 × 10^−6^ M. On the other hand, when the concentration is less than 1 × 10^−6^ M, the fluorescence intensity of tumor cells decreases sharply or disappears. To analyze the detection time, the TPN-labeled A549 was placed in the PBS at room temperature and assayed for fluorescence within 12 h. The fluorescence intensity of the TPN gradually decreased over time (Figure 5A). Therefore, the detection of cell fluorescence should be initiated after the TPN labeling of cells.

After that, MCF-7 and Hela were added to the tumor cell group, and their fluorescence intensity was measured to further prove the feasibility of a TPN concentration of 1 × 10^−6^ M. The statistical analysis (Figure 5B) and images (Figure 5C) showed that there was a significant difference in the fluorescence intensity between the tumor cell lines and the non-tumor cell lines (*p* < 0.001).

### 3.4. Investigation of the Performance of TPN-Labeled Cells on a Microfluidic Chip

**Figure 6:**
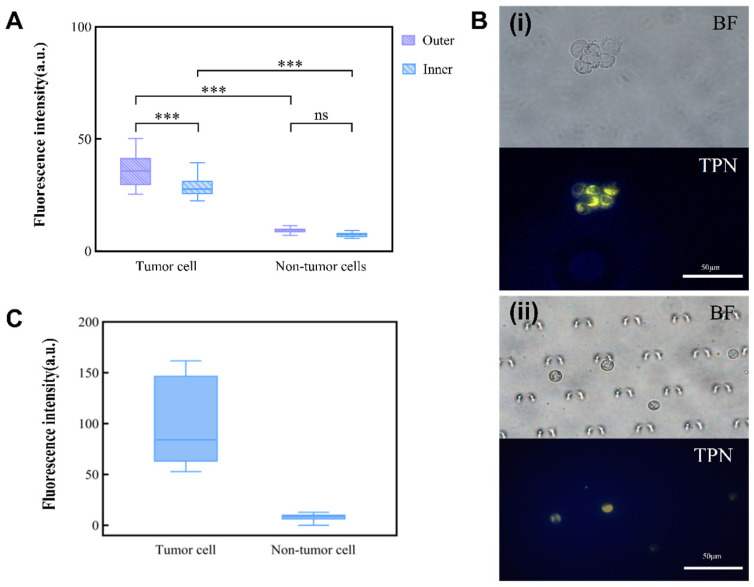
Influence of chip environment on TPN fluorescence. (**A**) Inner and outer chip analysis of fluorescence difference between tumor and non-tumor cells. (**B**) (**i**) TPN-labeled tumor cells before injection into the microfluidic chip; (**ii**) tumor cells captured in the microfluidic chip (BF, bright field; TPN, fluorescence field). (**C**) Fluorescence intensity of TPN-labeled pleural effusion cells (*** *p* < 0.001, Statistical insignificance was defined as ns).

The foundation for the integrated detection of an MPE on a chip is the observation of TPN-labeled cells on the microfluidic chip. TPN-labeled tumor cells (A549) and non-tumor cells (MET-5A) were injected separately into the chip for this purpose. As the non-tumor cells were negative, there was no statistically significant difference in the fluorescence intensity of the TPN-labeled MET-5A inside and outside the chip. However, the fluorescence of A549 showed a significant tendency to decrease (Figure 6A). Nevertheless, a comparison of the fluorescence between A549 and MET-5A within the chip showed that they remained significantly different (*p* < 0.001) (Figure 6B). This suggests that after concentration by the microfluidic chip, the cultured cell lines (A549 and MET-5A) can still be differentiated by TPN.

### 3.5. TPN Marking of Cells in Clinical Pleural Effusions

**Figure 7:**
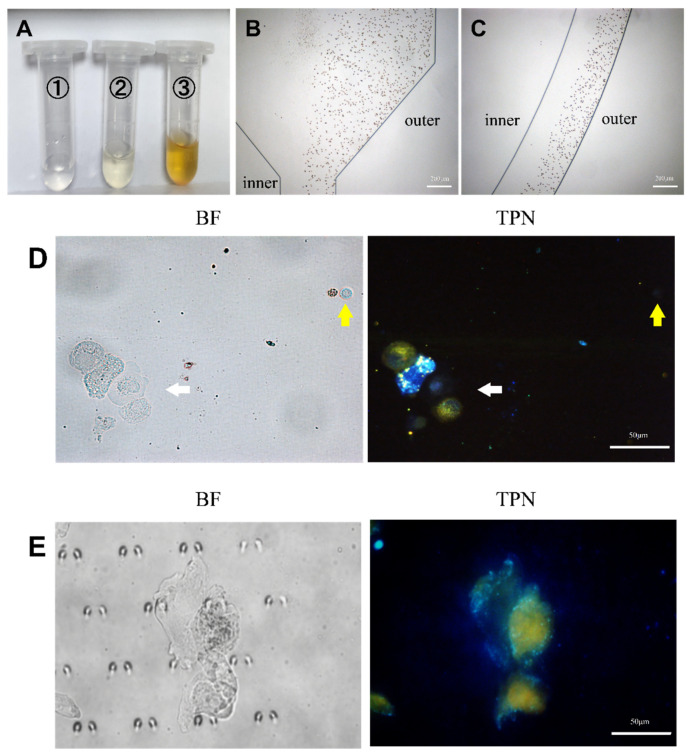
Analysis of pleural effusion. (**A**) The pleural effusion was processed by the microfluidic device and collected from the microfluidic outlet (①, ②, and ③). (**B**,**C**) Trajectories of RBCs in the microfluidic chip. (**D**) TPN-labeled pleural effusion cells before chip processing. The white arrow indicates the cluster of tumor cells in pleural effusion. The yellow arrow indicates WBC with RBC to the left, and both were fluorescence-negative. (**E**) Tumor cells captured on the microfluidic chip (BF, bright field; TPN, fluorescence field).

Each pleural effusion included in this study was divided into two parts: one part was subjected to an HE staining of the pleural effusion cells, while the other part labeled the cellular mitochondrial membranes with TPN. Cell types and proportions in the sample were counted by HE staining prior to processing the cells on the chip (Figure S4 and Table S1). The cell morphology and fluorescence performance were observed simultaneously using fluorescence microscopy, and the cell type was confirmed based on the cell morphology. A cluster of tumor cells was found in the pleural effusion, which showed positive TPN labeling, while the rest of the non-tumor cells were negative (Figure 7D). A gray-scale analysis of the labeled cells showed that the fluorescence of tumor cells is significantly stronger than that of non-tumor cells, which do not fluoresce (Figure 6C).

The pleural fluid was then used to verify the analytical conditions of the microfluidic chip that was obtained by culturing the cells as described in the previous section. Figure 7A displays the amount of waste fluid collected at the chip outlet. The erythrocytes in the pleural effusion were hydrodynamically guided to the outside of the microchannel track of the chip (Figure 7B,C) to be discharged through Outlet 3. Details can be obtained from Figure S3. It is not difficult to find that most of the erythrocytes are excluded from the chip through Outlet 3, and the rest of the erythrocytes that do not have time to enter the outer track mostly leave the chip through Outlet 2. The cells enriched by the capture area of the chip appeared as yellow fluorescent and were thought to be tumor cells (Figure 7E). In addition, each confirmed tumor cell was scored by a combination of three cytopathologists with extensive clinical experience in identifying pleural effusion tumor cells. The results demonstrate the feasibility of the method described in this study for clinical applications in pleural effusions.

With the development of microfluidic microarray technology, composite microarrays consisting of a combination of multiple single-track structures are increasingly being used for the analysis of clinical samples. Microfluidic technology combined with computer technology to facilitate the diagnosis and research of diseases is promoting microfluidics in medical automation [33]. The microfluidic chip is controlled by a computer to further minimize the impact of human manipulation on diagnostic results [34]. In the research of the diagnosis of pleural effusions, image recognition should be used for effusion cells to improve the diagnosis rate of malignant effusions [35].

## 4. Conclusions

In this study, we presented an integrated microfluidic device for separating and concentrating tumor cells in pleural effusions. The results show that the purity of tumor cells enriched by the microfluidic chip could be 98.7–99.3%. Meanwhile, an integrated microfluidic chip was realized to capture and detect TPN-fluorescence-labeled cells, which has the potential of specifically identifying tumor cells in clinical pleural effusions.

## Figures and Tables

**Table 1:** The ability of microarrays to concentrate on tumor cells.

Projects	A549	MCF-7	Hela
Purity (%)	94.1~96.8	98.5~99.3	98.1~98.7
Purity fold (X)	3.15~3.23	3.28~3.31	3.27~3.29

## Data Availability

The original contributions presented in the study are included in the article, further inquiries can be directed to the corresponding author.

## Supplementary Materials

The following supporting information can be downloaded at: https://www.mdpi.com/article/10.3390/mi15080981/s1, Figure S1: Parameters for the chip. Figure S2. The effect of cells being separated after cell-suspension injection into the microfluidic chip. Figure S3. Cells running in chip channels. Figure S4. Hematoxylin–eosin (HE)-staining images of cells in pleural effusion. Table S1. Sample Information.
