# Supplementary material for: Research on the Method of Detecting TPN-Labeled Tumor Cells in Pleural Effusion Based on the Microfluidic Chip
Source: Micromachines (Basel). 2024 Jul 30;15(8):981. doi: 10.3390/mi15080981 (PMC11356568; doi:10.3390/mi15080981)
Supplement: Supplementary file 1 [file micromachines-15-00981-s001.zip › micromachines-3107346-supplementary.pdf]

micromachines

MDPI

Supplementary Materials

Research on the Method of Detecting TPN-Labeled Tumor Cells in Pleural Effusion Based on the Microfluidic Chip

Xiaoyi Xun [1,†], Shuang Song [2,3†], Yiran Luan [1], Xiaoyue Long [4], Peilan Zhang [4], Yuqun Zheng [1] and Xuguo Sun [1,*]

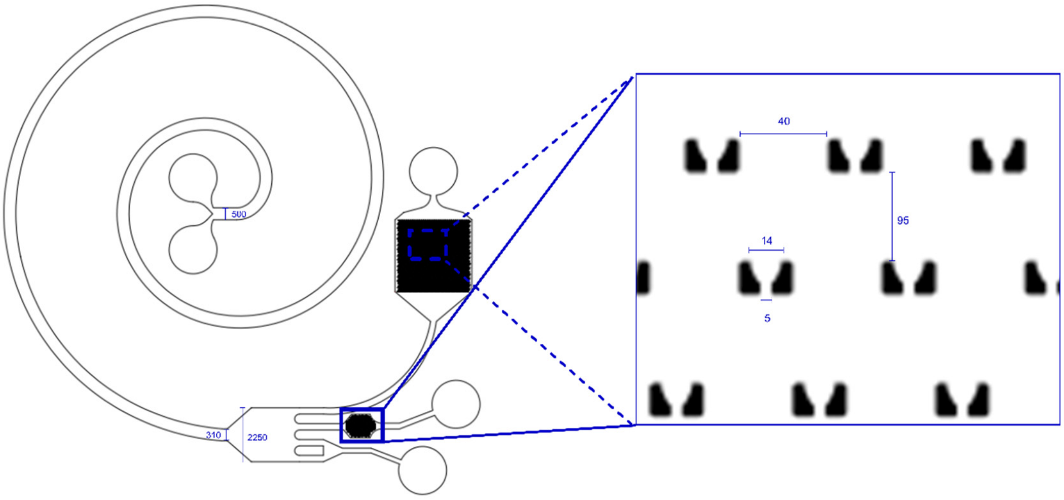

Figure S1. Parameters for the chip (Unit:μm). Except for the entrance, where the channel width is 500, the width of the spiral channel is 310μm. The width of the expansion channel connecting the spiral channel is 2250μm. There were 2310 trapping sites in capture area 1 and 273 trapping sites in capture area 2. Horizontal distance between trapping sites is 40μm, while the vertical distance is 95μm. Trapping site has a top diameter of 14μm and a bottom diameter of 5μm.

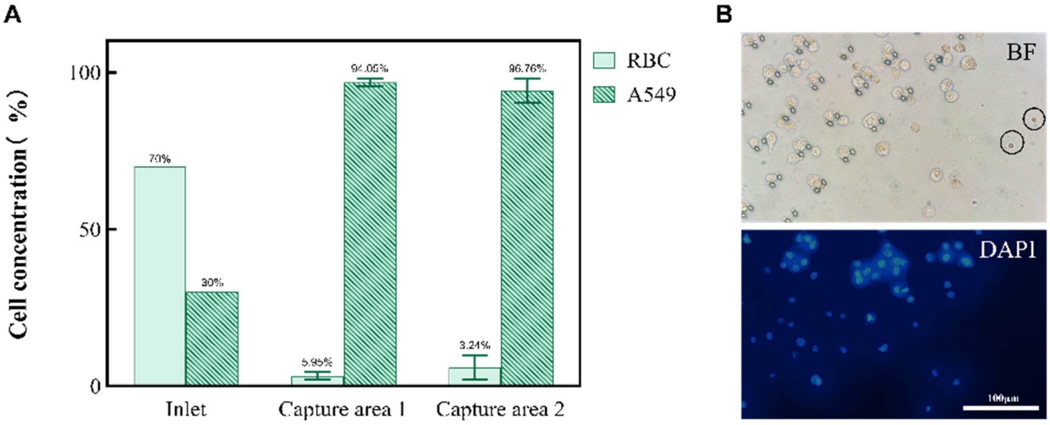

Figure S2. The effect of cells being separated after cell suspension injection into the microfluidic chip. (**A**). Changes of tumor cells (A549) and RBCs in the capture areas before and after the cell suspension were processed by the microarray. (**B**) Tumor cells (A549) were trapped in the capture areas and rare RBCs (indicated by black circles in the bright field) were mixed into there. Microscopic images include bright field (BF) and fluorescence field (DAPI).

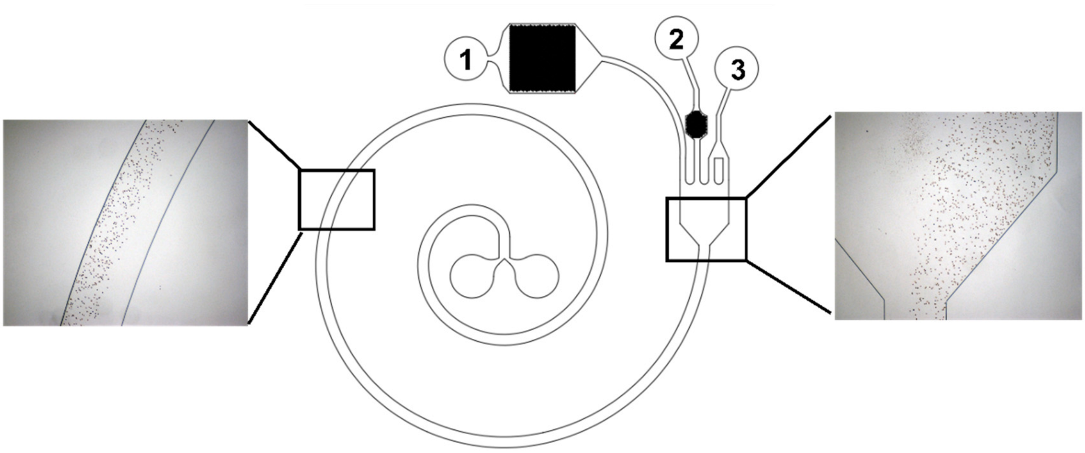

Figure S3. Cells running in chip channels. ① ② ③ are collected separately from the liquid at the outlet of the 1.2.3.

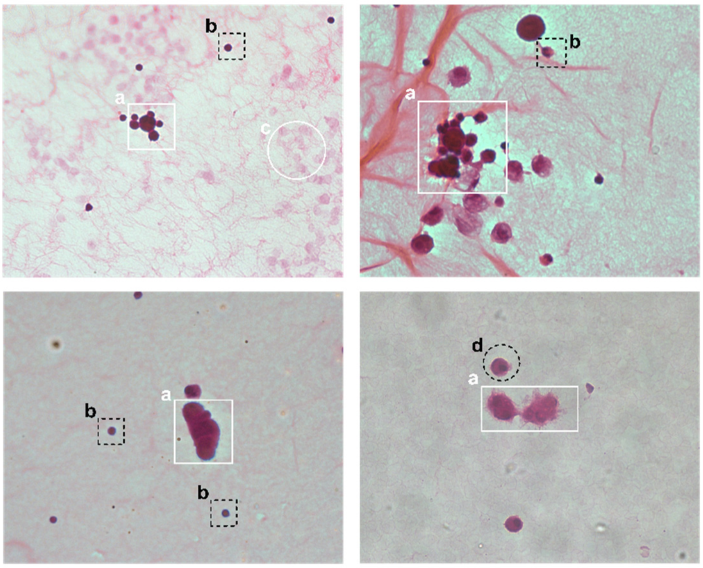

Figure S4. Hematoxylin-eosin (HE) staining images of cells in pleural effusion. a. Tumor cells. b. White blood cells. c. Red blood cells. d. Epithelial cells.

Table S1. Sample Information.

Sample No.	Diagnosis	Color	Clarity	Leukocytes($10^9/L$)	Monocytes(%)	Multinucleated cells(%)	Mesothelial cells/Nucleated Cells
1	Lung cancer	Orange peeling	Cloudy	0.6	83	17	5/17
2	Lung cancer	Orange peeling	Cloudy	0.7	50	50	5/19
3	Lung cancer	Yellowish	Clear	1	97	3	3/22
4	Lung cancer	Red	Cloudy	0.6	75	24	2/56
5	Adenocarcinoma of the lung	Orangish	Cloudy	0.5	90	10	0
6	Lung Cancer Post-Operative	Orange peeling	Cloudy	2	90	10	2/42
7	Right lung cancer	Yellowish	Cloudy	1.6	95	5	2/34
8	Lung cancer	Yellow	Cloudy	5	73	27	6/106
9	Lung Cancer Post-Operative	Orange peeling	Cloudy	3.7	100	0	0/74
10	Lung cancer	Orange	Cloudy	0.2	0	0	2/10
11	Mesothelioma	Orange	Cloudy	7.2	10	90	3/100
12	Lung cancer	Coffee	Cloudy	1	10	90	2/22
13	Lung cancer	Milky	Cloudy	2.2	80	20	1/45
14	Lung cancer	Yellow	Clear	0.75	100	0	2/17
15	Lung cancer	Milky	Cloudy	0.4	0	0	2/10
